# What is your diagnosis?

**DOI:** 10.4274/jtgga.2017.0117

**Published:** 2018-03-01

**Authors:** Vatsla Dadhwal, Latika Chawla, Aparna K. Sharma, Dipika Deka

**Affiliations:** 1 Department of Obstetrics and Gynecology, All India Institute of Medical Sciences, New Delhi, India

A 25-year-old, second gravida woman was referred to the fetal medicine clinic at 19 weeks’ gestation for a fetal skin biopsy. Her first baby was a home delivery and had abnormally thick, rough skin and expired on day 2 of life. Two previous ultrasound scans in this pregnancy at 16 weeks and 18 weeks were reported to be normal. On repeating the ultrasound, we saw clenched fists, contractures of toes, and minimal fetal movement with stiff limbs in a semi-flexed position. A 3-dimensional (3D) scan showed thick pouting lips, flat nasal bridge, distal contractures of the toes with incurving, clenching of fingers, and flattened ears (Figure 1). Taking into account the history of consanguinity, a previous baby with a skin disorder, and these ultrasound findings, the parents were counseled and after deliberation, they opted for termination of the pregnancy. A postnatal examination confirmed the features seen on 3D ultrasound (Figure 1). The fetus showed facial dysmorphism, clenched fists, and contractures of the toes of both limbs. A histopathologic examination of the skin biopsies (from the fetal forearm, trunk, lower limbs, and buttocks) revealed marked orthokeratotic hyperkeratosis with a granular layer.

Four years later, the woman conceived again and consulted us at 18 weeks’ gestation. Unfortunately, even in this pregnancy, the fetus on 2D and 3D ultrasound was found to have similar features (Figure 2) as observed in her last pregnancy; therefore, after counseling, the pregnancy was terminated. Post-natal examination of the fetus corroborated the findings seen on the 3D scan (Figure 2).

## Answer

On the basis of the characteristic phenotypic features of the first born who succumbed and ultrasound findings in second pregnancy, a suspicion of ichthyosis was entertained, the couple was counseled, and they agreed for the termination of pregnancy. Post abortion, fetal skin biopsy confirmed the diagnosis. Also in the third pregnancy, diagnosis was made on ultrasound because the couple could not afford a molecular prenatal diagnosis.

Ichthyosis is described as a group of skin disorders of keratinization, characterized by generalized scaling of the skin with varying severity. The great majority are inherited. The molecular basis and pathophysiology of most inherited ichthyosis is described with the identification of mutations in gene coding for various proteins or enzymes involved in a broad variety of cellular functions from DNA repair to skin barrier homeostasis. They vary from less severe to more severe forms. The mode of inheritance is autosomal semi-dominant, X-liked or recessive (1). Harlequin ichthyosis (HI), the most severe form, is an extremely rare autosomal recessive skin disorder that is almost always fatal in the early days of life (2). If both parents are carriers for genetic mutation, there is a 25% chance of each pregnancy being affected. It was unfortunate that 3 consecutive pregnancies were affected in our case. The skin is shiny, white, thick, with hyperkeratotic plaques and polygonal or diamond shaped fissures/cracks (3). The skin thickening results in clown-like faces with an eclabium (eversion of lip), ectropion (eversion of eyelids), a hypoplastic nose, small rudimentary/absent ears, and contractures in the upper and lower limbs with incurved toes and clenched fists (4). The abnormal keratinization of the skin leads to dehydration and infection as a result of the defective skin barrier, from which newborns succumb. Mutations on the Adenosine Triphosphate Binding Cassette Transporter Protein A12 (ABCA12) gene, a keratinocyte lipid transporter, lead to this condition (5).

Prenatal diagnosis of HI was first described in 1983 by Blanchet-Bardon et al. (6) in a couple with a previous harlequin birth, through fetal skin biopsy at 20-22 weeks. Fetal skin biopsy between 17-22 weeks remained the standard of care for prenatal diagnosis till recent times, with pathologic analysis consistent with accelerated hyperkeratosis (7). There have been reports of cytologic analysis of centrifuged amniotic fluid showing the same pathology (8). The isolation of the ABCA12 gene has made prenatal diagnosis possible early in pregnancy through chorionic villous sampling and amniocentesis with the option of terminating affected fetuses (9,10). Attempts have been made to make a diagnosis on ultrasound, mostly late in the second and third trimester. Unfortunately, medical termination of pregnancy cannot be offered after 20 weeks’ gestation in Turkey.

Many authors have used ultrasound to make a diagnosis by looking at the characteristic features as described above. Mihalko et al. (2) were probably the first to report suggestive findings on 2D-ultrasound at 28 weeks’ gestation but did not make a prenatal diagnosis. They reported the presence of a thick discontinuous membrane floating in front of the fetus, restricted fetal movements, masses anterior to each orbit, and a thickened scalp.

Bongain et al. (11) were amongst the first to use 3D-ultrasound for the diagnosis of HI in a woman in two pregnancies. In the first pregnancy, diagnosis was made at 30 weeks when the woman was referred with an abnormal ultrasound and the pregnancy was terminated without a prenatal diagnosis because characteristic features were seen on 2D/3D scans. In the second pregnancy, thick lips and echogenic liquor were noted at 17 weeks. At 22 weeks, on 3D-ultrasound, an open mouth and thick lips were observed and a fetal skin biopsy confirmed the diagnosis of HI. Holden et al. (12) described another case with distal arthrogryposis at 24 weeks in a couple with a previously affected child. Flattening of the facial profile was noted only at 32 weeks.

Ultrasound diagnosis can be challenging in the index case. This has been highlighted in case reports with reports of typical findings on 2D/3D scans without a primary diagnosis of HI and confirmation of the diagnosis only after delivery (13,14).

This case report is one of the very few that shows accurate early detection (before 20 weeks’ gestational age) using ultrasound without invasive testing. All the typical facial features, clenched hands, and toe contractures were appreciated on 2D and 3D-ultrasound. It highlights the fact that in centers where molecular diagnosis/fetal skin biopsy is unavailable, it is possible to use 3D-ultrasound in addition to 2D-ultrasound as a useful tool in clinching the diagnosis, especially in couples with a previously affected child. 3D images have a photograph-like realism, especially when looking at typical “fish-like facial morphology”. This gives confidence in making a diagnosis in the absence of skin biopsy or molecular prenatal diagnosis. It also emphasizes the fact that diagnosis in a high-risk case requires serial ultrasound monitoring for suspicion of these subtle features because they may not develop at the routine anomaly screening at 18-20 weeks. There have been a few case reports of diagnoses of skin fissures/cracks in other types of ichthyosis on ultrasound imaging (15).

In conclusion, the availability of molecular testing without doubt simplifies the process of prenatal diagnosis of HI. However, with the non-availability of molecular testing/skin biopsy, careful and serial 2D/3D- ultrasound by experienced sonographers can achieve early diagnosis without resorting to invasive testing in couples with a previously affected child.

## Figures and Tables

**Figure 1:**
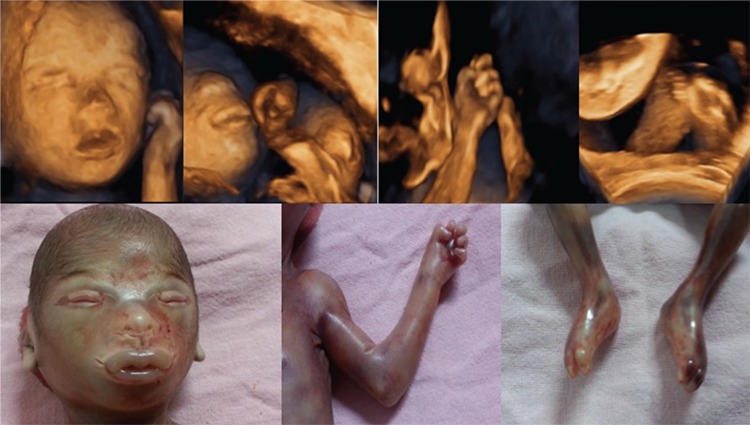
Antenatal 3-dimensional-ultrasound images and post-natal images of the fetus in the second pregnancy showing the typical phenotypic features of Harlequin ichthyosis (eclabium, ectropion, small nose, clenched fists and incurved toes)

**Figure 2:**
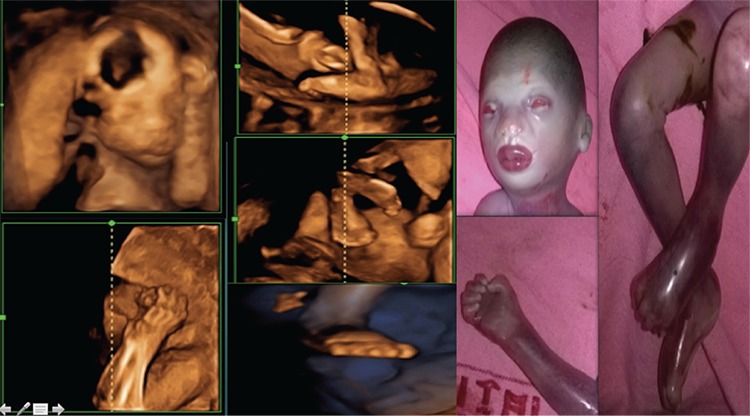
Antenatal 3-dimensional-ultrasound images and post-natal images of the fetus in the third pregnancy showing the classic phenotypic features of Harlequin ichthyosis (eclabium, ectropion, small nose, clenched fists, incurved toes and contracture at the bilateral knee joints)
